# Use of the Multidimensional Haemophilia Pain Questionnaire (MHPQ) in Patients With Severe Haemophilia A Treated With Emicizumab: A Pilot Cross‐Sectional, Single‐Centre Study

**DOI:** 10.1111/hae.70318

**Published:** 2026-05-17

**Authors:** Ilenia Lorenza Calcaterra, Sofia Donnarumma, Guido D'Errico, Carmine De Luca, Chiara Caputo, Paola Ciciola, Ernesto Cimino, Matteo Di Minno

**Affiliations:** ^1^ Department of Clinical Medicine and Surgery, Regional Reference Centre For Coagulation Disorders Federico II University Naples Italy

**Keywords:** arthropathy, chronic pain, Emicizumab, haemophilia A, MHPQ, multidimensional pain assessment, pain interference, patient‐reported outcomes

## Abstract

**Background:**

Despite effective bleed prevention with Emicizumab, pain remains a relevant clinical burden in adults with severe haemophilia A (sHA). A multidimensional assessment is needed to better characterize contemporary haemophilia‐related pain.

**Objective:**

To evaluate the prevalence, characteristics, and functional impact of pain in adults with sHA receiving Emicizumab prophylaxis using the Multidimensional Haemophilia Pain Questionnaire (MHPQ).

**Methods:**

In this single‐centre, cross‐sectional pilot study, 27 adults with sHA on Emicizumab prophylaxis completed the MHPQ. Pain localization, frequency, triggering factors, intensity, and interference across life domains were assessed descriptively.

**Results:**

Lifetime haemophilia‐related pain was reported by 85.2% of participants, and 77.8% experienced pain within the previous 12 months. Pain most frequently involved ankles (52.4%), knees (52.4%), and elbows (28.6%). All patients with recent pain reported pain during bleeding episodes; however, movement‐related triggers, including accidental movements (66.7%) and physical activity (54.2%), were also common. Pain intensity was generally mild‐to‐moderate, with the highest median scores observed after accidental movements (median 4, IQR 2–6). Pain interfered predominantly with general activity (71.4%), walking ability (66.7%), mood (61.9%), and enjoyment of life (61.9%). Arthropathy was documented in 70.4% of patients.

**Conclusions:**

In the non‐factor prophylaxis era, pain remains prevalent and functionally relevant in adults with sHA despite effective bleed control. These findings suggest that haemophilia‐related pain is not exclusively bleeding‐driven and highlight the importance of structured, multidimensional pain assessment in routine haemophilia care.

## 1 Introduction

Haemophilia A is an X‐linked inherited bleeding disorder caused by a deficiency of coagulation factor VIII (FVIII), leading to an increased risk of spontaneous bleeding, particularly within joints (haemarthroses) [1].

Recurrent hemarthroses trigger a cascade of inflammatory and degenerative changes, ending in haemophilic arthropathy [2, 3]. Haemophilic arthropathy, defined as the chronic joint disease affecting PwH leading to synovial inflammation, cartilage and bone damage [4], is a common major complication leading to disability, chronic pain, and impaired quality of life [5]. Despite the advent of effective prophylactic FVIII replacement therapies, haemophilic arthropathy persists in approximately 25%–30% of patients, underscoring the need for continuous awareness in joint health management [1]. Pain remains a prevalent and debilitating symptom among people with haemophilia (PWH), manifesting acutely during bleeding episodes and chronically due to established arthropathy [6]. The coexistence of acute and chronic pain presents unique challenges in assessment and management, often leading to suboptimal treatment outcomes [6, 7].

Acute and chronic pain are experienced by many PWH: in a 2013–2014 study of 381 males with haemophilia and a history of joint pain/bleeding, 20.0% had experienced acute pain, 34.0% had experienced chronic pain and 32.0% had experienced both acute and chronic pain in the last 6 months [8].

Emicizumab, a recombinant, humanized bispecific monoclonal antibody, received FDA approval in 2017 for prophylactic use in haemophilia A. By bridging activated factor IX and factor X, Emicizumab mimics the function of activated FVIII, thereby restoring haemostatic balance [9, 10]. As of June 2023, more than 24,000 people have been treated with Emicizumab globally [11]. Clinical trials have proved Emicizumab efficacy and safety across various patient populations, including those with and without FVIII inhibitors, and spanning all age groups [12].

Notably, Emicizumab prophylaxis has been shown to significantly reduce bleeding rates, particularly joint bleeding, and to improve overall joint outcomes in patients with haemophilia A [11]. Given the close relationship between joint bleeding, synovial inflammation, and pain development, these clinical benefits provide a strong biological rationale for a potential impact of Emicizumab on pain burden. However, pain‐related outcomes have not been consistently or systematically evaluated in Emicizumab clinical trials, and direct evidence on both acute and chronic pain remains limited [13]. Therefore, the effect of Emicizumab prophylaxis on multidimensional pain experiences warrants further investigation.

Previous studies in patients receiving conventional FVIII prophylaxis have reported persistent acute and chronic pain despite effective bleeding prevention, highlighting the complexity of pain mechanisms beyond bleeding control alone [5, 6, 7].

Given the multifactorial nature of pain in haemophilia and the limitations of existing assessment tools, the Multidimensional Haemophilia Pain Questionnaire (MHPQ) [14] was developed to provide a comprehensive evaluation of pain experiences in this population. Specifically designed to capture haemophilia‐specific pain features, including the coexistence of acute and chronic pain, multiple painful locations, and functional triggers, the MHPQ represents a valuable tool for multidimensional pain assessment [14]. This pilot study aims to explore multidimensional pain profiles in PWH A receiving Emicizumab prophylaxis.

## 2 Materials and Methods

### 2.1 Study Participants

This single‐centre, observational, cross‐sectional pilot study received approval from the local Ethics Committee of the University of Naples Federico II. The study design adhered to the STROBE (Strengthening the Reporting of Observational Studies in Epidemiology) guidelines for observational studies [15]. Consecutive patients with severe haemophilia A (sHA), defined by factor VIII (FVIII) activity levels <1%, were screened for eligibility during clinical evaluation during scheduled follow‐up. The inclusion criteria were diagnosis of sHA (FVIII <1%); Age ≥18 years, Ongoing prophylactic treatment with Emicizumab for a minimum of 6 months; Ability to provide informed consent. The exclusion criteria were: Active infections with hepatitis B virus (HBV), hepatitis C virus (HCV), or human immunodeficiency virus (HIV); Diagnosed rheumatologic diseases; Concurrent treatment with anticoagulant or antiplatelet medications; Presence of bleeding disorders other than haemophilia A; Presence of clinical signs of acute joint bleeding or documentation of such an episode within two weeks prior to enrolment.

Upon obtaining informed consent, demographic and clinical data were collected, including age, body mass index (BMI), baseline FVIII activity (confirming sHA), annual bleeding rate (ABR), annual joint bleeding rate (AjBR), history of haemophilic arthropathy. All patients included in the study receive regular follow‐up at our Haemophilia Treatment Centre, where standardized joint clinical evaluations and point‐of‐care ultrasound assessments are performed as part of routine care. The presence or absence of haemophilic arthropathy was determined based on consolidated medical records.

Haemophilic arthropathy was defined as a documented diagnosis of chronic haemophilic joint disease established by the treating physicians, based on clinical examination following the HJHS 2.1 protocol and ultrasound findings assessed according to the HEAD‐US score. For this analysis, arthropathy was treated as a binary variable (present/absent), and patients were classified as affected if at least one joint was clinically diagnosed as arthropathic.

### 2.2 Pain Assessment

Pain was evaluated using the MHPQ [14], a validated patient‐reported outcome measure developed within a biopsychosocial framework and aligned with the Initiative on Methods, Measurement, and Pain Assessment in Clinical Trials (IMMPACT) recommendations [16]. The MHPQ was specifically designed to capture the distinctive features of haemophilia‐related pain, including the coexistence of acute and chronic pain, the presence of multiple painful locations, and the functional and psychosocial impact of pain.

The questionnaire opens with four preliminary screening items designed to distinguish acute from chronic pain, based on criteria proposed by the European Haemophilia Therapy Standardization Board, namely pain lasting longer than three months and occurring more than once per week. If no haemophilia‐related pain is reported in the previous 12 months, the remaining sections of the questionnaire are not completed. For patients reporting pain within the last year, all subsequent questions refer to that time frame.

The MHPQ evaluates nine dimensions of haemophilia‐related pain, each analysed independently, as no overall composite pain score is calculated. Pain localization is assessed by allowing participants to identify all painful joints and to specify both the most painful location and the site causing the greatest functional impact. Pain duration is explored through an open‐ended question regarding the onset of the most impactful pan. Pain frequency and temporal pattern are assessed by enquiring about how often pain occurs (e.g., during bleeding episodes, weekly, daily, continuously), daily timing of peak pain intensity, and the most recent pain episode.

Triggering factors are identified through a predefined list, including bleeding episodes, physical effort or movement, stair climbing, accidental or ‘wrong’ movements, weather changes, and pain occurring at rest. Pain intensity is measured using a 0–10 numerical rating scale (NRS), where 0 indicates ‘no pain’ and 10 indicates ‘worst imaginable pain’. Intensity is rated under six specific conditions corresponding to the triggering contexts described above. An average intensity score can be computed by calculating the mean of these six items, yielding a global intensity score ranging from 0 to 10, with higher values reflecting greater perceived pain severity.

Pain interference is assessed using the interference items derived from the Brief Pain Inventory, evaluating the extent to which haemophilia‐related pain interferes with seven domains: general activity, mood, walking ability, normal work, relations with other people, sleep, and enjoyment of life. Each item is rated on a 0–10 NRS, where 0 indicates ‘does not interfere’ and 10 indicates ‘completely interferes’. A mean interference score can be calculated across items, resulting in a global interference score ranging from 0 to 10, with higher scores indicating greater functional and psychosocial impact.

Pain management strategies are assessed by asking participants to select pharmacological and non‐pharmacological approaches used to manage pain (e.g., analgesics, clotting factor replacement, rest, ice, compression), and to rate the perceived relief obtained from each selected strategy on a 0%–100% scale, where higher percentages reflect greater perceived effectiveness. The questionnaire also includes items regarding consultation with pain management specialists and overall satisfaction with pain treatment, the latter rated on a 5‐point Likert scale ranging from ‘very dissatisfied’ to ‘very satisfied’ [14].

### 2.3 Statistical Analysis

Descriptive statistics were used to summarise the study population and MHPQ outcomes. Continuous variables are reported as mean ± standard deviation or median (interquartile range, IQR), as appropriate. Categorical variables are reported as counts and percentages.

Given the exploratory nature and limited sample size, no formal hypothesis testing was prioritised; analyses were primarily descriptive. All statistical analyses and graphical representations were performed using R software (R Foundation for Statistical Computing, Vienna, Austria; version 4.5.2) within the RStudio environment.

## 3 Results

A total of 27 adults with sHA receiving Emicizumab prophylaxis completed the MHPQ. Mean age was 48.0 ± 14.3 years; haemophilic arthropathy was documented in 19 patients (70.4%), and four (14.8%) had a history of FVIII inhibitors, before Emicizumab initiation, 22.2% of patients achieved a zero ABR, compared with 59.3% after treatment initiation. (Table 1, 2).

**TABLE 1:** Demographic and clinical characteristics of the study population.

	Study population (*n* = 27)
Age (years)	48.0 ± 14.3
Severe haemophilia (%)	100%
Male (%)	100%
Inhibitor carriers (%)	14,8%
Duration of Emicizumab prophylaxis (months), median (IQR)	12.0 (12.0–24.0)
Number bleeding/year pre‐Emicizumab prophylaxis median (IQR)	2.0 (1.0–4.0)
Number bleeding/year post‐Emicizumab prophylaxis (median (IQR)	0.0 (0–1.8)
Number hemarthrosis/year pre‐Emicizumab prophylaxis median (IQR)	2.0 (1.0–3.0)
Number hemarthrosis/year post‐Emicizumab prophylaxis median (IQR)	0.0 (0–1.0)
Weight (Kg)	85.1 ± 20.1
Prevalence of haemophilic arthropathy (%)	70.4 %
Patients with 0 bleeding/year pre‐Emicizumab	6 (22.2%)
Patients with 0 bleeding/year post‐Emicizumab	16 (59.3%)

*Note*: ABR and AjBR were derived from routine clinical records collected during standard follow‐up visits and are reported as median (IQR) for the 12 months preceding and following initiation of Emicizumab prophylaxis. Duration of Emicizumab refers to time on treatment at the time of pain assessment. Inhibitor status indicates a documented history of FVIII inhibitors; no patients had active inhibitors at the time of study assessment.

**TABLE 2:** Strategy adopted to manage pain.

Strategy adopted	Patients *N* (%)
Pain medication	12 (57.1%)
Compression	11 (52.4%)
Ice	10 (47.6%)
Tobacco or recreational drugs	9 (42.9%)
Meeting friends	6 (28.5%)
Rest	6 (28.5%)
Clotting factor replacement	5 (23.8%)
Therapeutic massage	5 (23.8%)
Other^a^	8 (38.2%)

^a^ Includes: relaxing techniques, meditation, homeopathy

**FIGURE 1:**
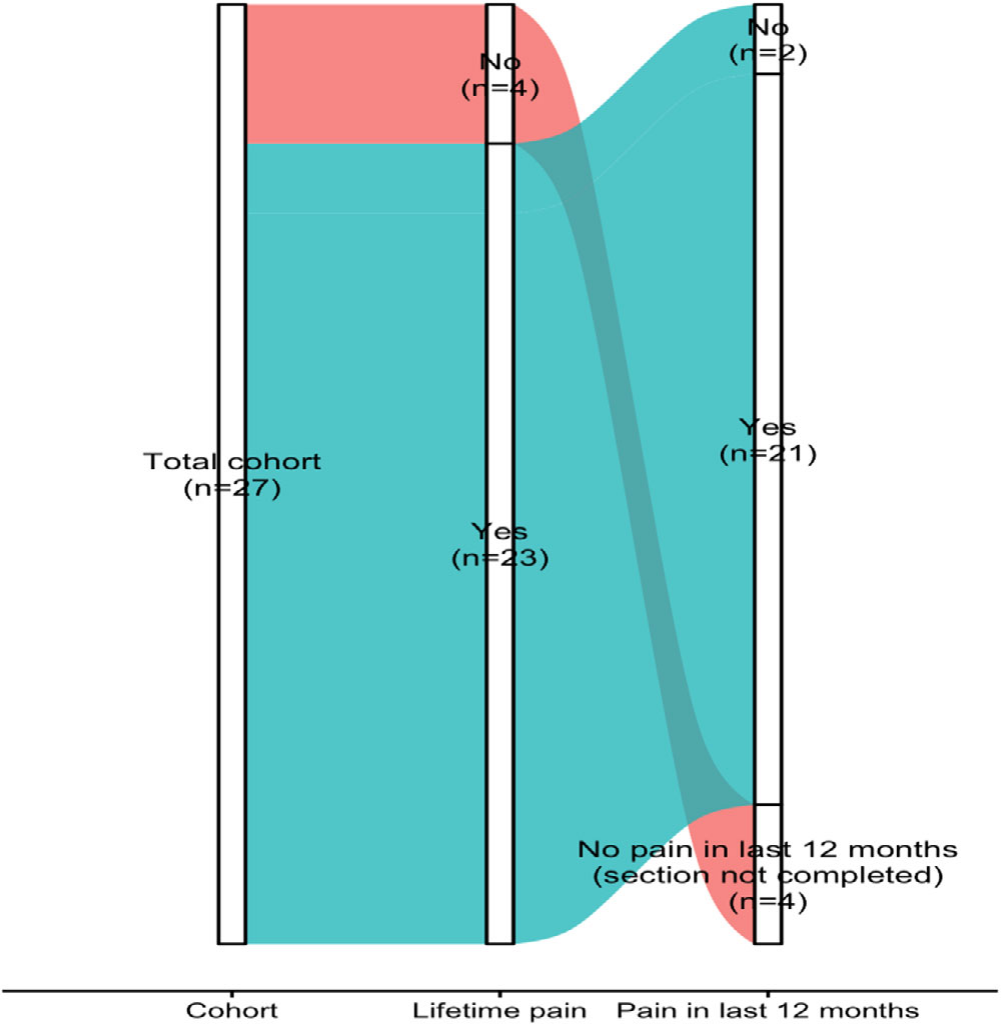
Flow diagram showing the distribution of haemophilia‐related pain in 27 adults with severe haemophilia A receiving Emicizumab prophylaxis. Patients who did not report pain within the last 12 months did not complete subsequent MHPQ sections, as per questionnaire structure.

**FIGURE 2:**
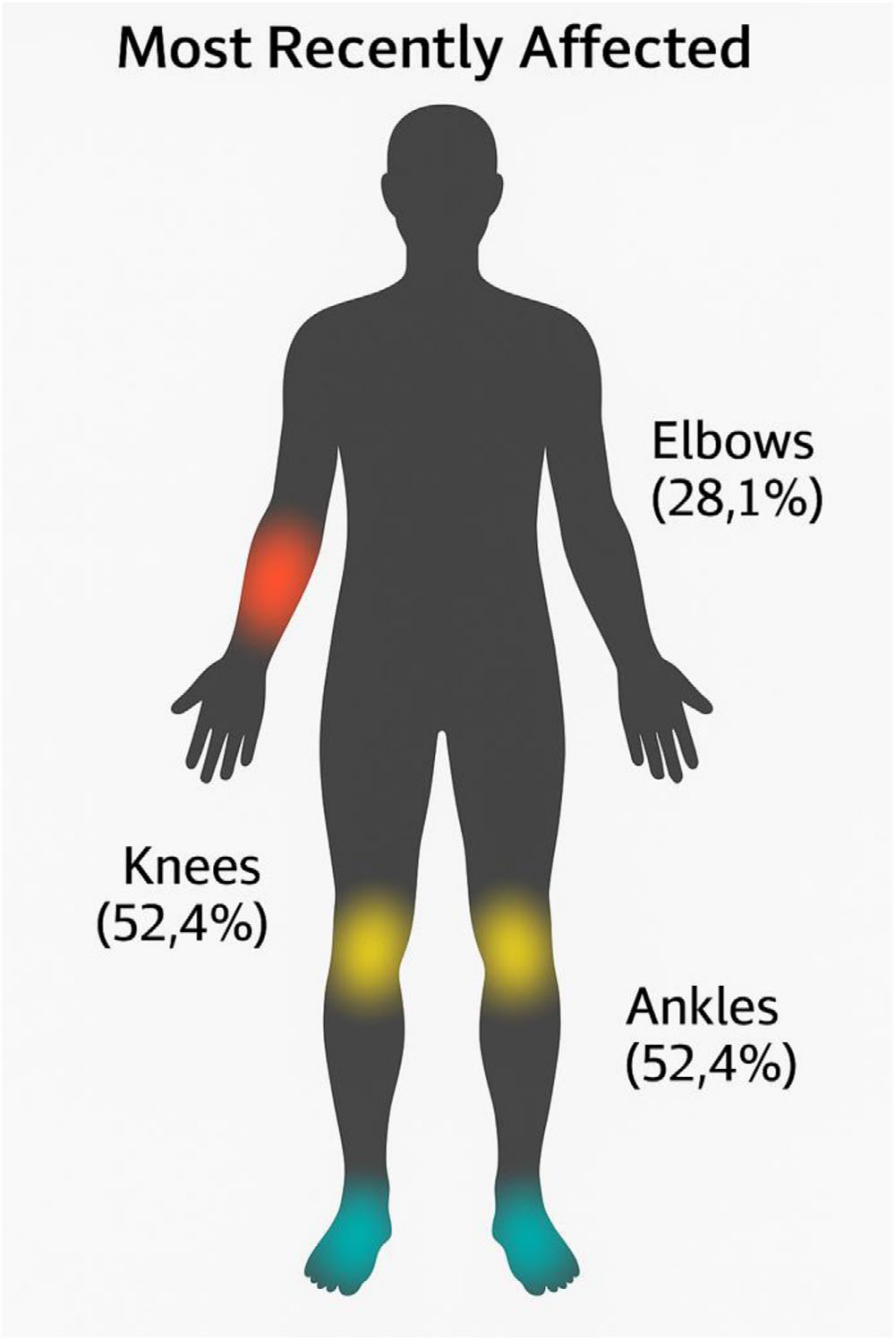
Anatomical heatmap showing the joints most recently affected by haemophilia‐related pain, as reported by adult patients with severe haemophilia A.

Overall, 23 of 27 participants (85.2%) reported haemophilia‐related pain at some point in their lifetime. Among the overall cohort, 21 of 27 (77.8%) reported at least one pain episode within the previous 12 months. Consequently, the six patients who did not report pain during the last year (22.2%) did not complete the pain‐specific sections of the questionnaire (Figure 1). Despite a median ABR of 0 (IQR 0–1) after Emicizumab initiation, 77.8% of the overall cohort reported pain within the last 12 months. Among patients reporting pain in the last year (*n* = 21), ankles (52.4%) and knees (52.4%) were the most frequently involved joints, followed by elbows (28.6%) (Figure 2). Ankles were also most frequently identified as the most painful site (47.6%) and as the joint causing the greatest functional impairment (47.6%).

**FIGURE 3:**
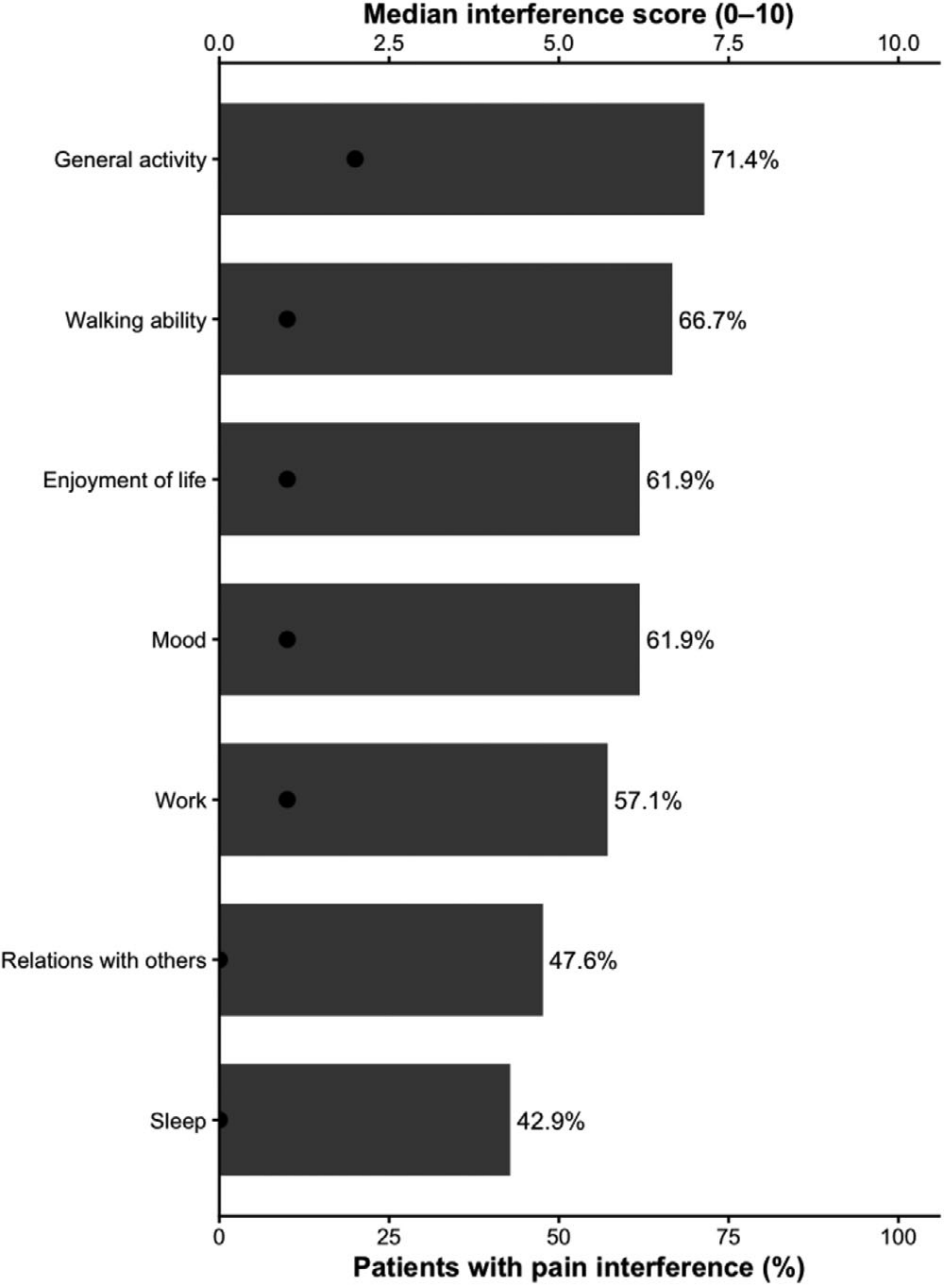
Pain interference profile across functional domains of the Multidimensional Haemophilia Pain Questionnaire (MHPQ) among patients reporting pain in the previous 12 months (*n* = 21). Bars represent the proportion of patients reporting interference in each domain, while points indicate the median interference score on a 0–10 numerical rating scale (secondary axis). General activity and walking ability were the most frequently affected domains.

The median duration of the most impactful pain was 8 years (IQR: 5–12). Regarding pain chronicity, 38.1% reported pain lasting longer than three months, while 9.5% reported pain occurring more than once per week. Pain during bleeding episodes was reported by all patients who experienced pain in the previous 12 months (21/21, 100%). Additional triggers included physical exertion or movement (54.2%) and accidental or ‘wrong’ movements (66.7%). Pain related to stair climbing and weather changes was reported by 23.8% of patients, while 14.3% experienced pain at rest. Pain was predominantly activity‐dependent (81%), with smaller proportions reporting worsening at the end of the day or during the night. Pain intensity was overall in the mild‐to‐moderate range. The highest median intensity was observed following accidental or ‘wrong’ movements (median 4; IQR: 2–6), followed by bleeding episodes (median 3; IQR: 2–5.5) and physical exertion (median 3; IQR: 1–6). Pain interference was reported across multiple functional domains (Figure 3). General activity was the most frequently affected domain (15/21; 71.4%; median 2), followed by walking ability (14/21; 66.7%; median 1), mood (13/21; 61.9%; median 1), and enjoyment of life (13/21; 61.9%; median 1). Work‐related activities were affected in 57.1% of patients, while interpersonal relationships (40.7%) and sleep (42.9%) were less commonly impacted. Median interference scores ranged from 0 to 2 across domains. The most commonly reported pain management strategies were analgesic use (57.1%), compression (52.4%), and ice application (47.6%). While 66.7% of participants reported being satisfied or very satisfied with their current pain management, 14.8% expressed moderate dissatisfaction (Table 2). Pain was more frequently reported among patients with documented HA compared with those without HA (63.2% vs. 37.5%), whereas no clear pattern emerged according to inhibitor history.

## 4 Discussion

This cross‐sectional pilot study explored the multidimensional pain experience of adults with sHA receiving Emicizumab prophylaxis using the disease‐specific MHPQ [14]. To our knowledge, this is among the first real‐world studies specifically characterizing multidimensional pain using a disease‐specific instrument in adults treated with Emicizumab. Beyond documenting pain prevalence, the primary contribution of this study lies in the detailed characterization of pain phenotype in the Emicizumab era. Despite substantial reductions in bleeding rates achieved with non‐factor prophylaxis, pain remains highly prevalent and functionally relevant, suggesting that haemostatic control alone does not fully address the complexity of haemophilia‐related pain. In our cohort, 85% of participants reported lifetime haemophilia‐related pain and nearly 78% experienced pain within the previous 12 months. These figures are comparable to those reported in earlier studies conducted in the context of FVIII prophylaxis [8], indicating that the global burden of pain has not disappeared with the introduction of Emicizumab. However, the multidimensional assessment provides additional insights into the qualitative nature of this residual pain.

Notably, pain in our population was most frequently triggered by accidental or ‘wrong’ movements and physical exertion, while lower intensity scores were reported at rest. Although pain during bleeding episodes was universally acknowledged, the prominence of movement‐related triggers suggests that the dominant pain phenotype in this treated population may be predominantly mechanical and functionally driven rather than exclusively haemorrhagic. In the era of non‐factor prophylaxis, haemophilia‐related pain may be undergoing a phenotypic transition, from predominantly bleeding‐driven acute pain toward a chronic, mechanically and biologically complex condition influenced by established joint damage, altered biomechanics, and central pain modulation mechanisms [17]. This evolving pain landscape requires assessment frameworks that extend beyond haemostatic metrics alone [17]. Recent evidence has further highlighted the complex biological and psychosocial mechanisms underlying haemophilia‐related pain [6].

The anatomical distribution of pain, predominantly affecting ankles and knees, is consistent with known patterns of haemophilic arthropathy [18]. However, contemporary literature increasingly reports discordance between imaging‐defined structural joint damage and patient‐reported pain intensity [19]. Within this framework, the persistence of pain despite effective bleed control should not be interpreted as treatment failure, but rather as evidence of the multifactorial nature of chronic pain in this population.

A key added value of this study is the use of the MHPQ, which enabled a multidimensional and disease‐specific characterization of pain. Unlike unidimensional scales that measure only intensity, the MHPQ captures pain chronology, triggers, temporal patterns, interference across life domains, coping strategies, and satisfaction with treatment. This approach revealed that even when intensity scores were moderate, pain interfered meaningfully with general activity, walking ability, mood, and enjoyment of life. Such findings underscore that pain burden cannot be fully appreciated by intensity measures alone and highlight the importance of assessing functional and psychosocial domains in routine clinical practice. Interestingly, while most patients reported satisfaction with their pain management, all individuals experiencing pain also reported some degree of interference. This apparent discrepancy may reflect adaptation phenomena or response shift, whereby patients recalibrate expectations over time in the context of chronic disease. From a therapeutic perspective, the predominance of on‐demand analgesic use and non‐pharmacological strategies suggests that pain management remains largely reactive and symptom‐driven. As bleeding frequency decreases under Emicizumab prophylaxis, comprehensive pain care may increasingly require multidisciplinary approaches addressing biomechanical factors, rehabilitation strategies, and psychosocial determinants.

Notably, studying a population treated with Emicizumab offers a unique opportunity to observe pain patterns in a context of markedly reduced bleeding burden. Compared with historical FVIII‐treated cohorts, the persistence of pain despite near‐zero ABRs suggests that residual pain may be more closely linked to chronic joint sequelae and altered pain modulation mechanisms rather than ongoing haemorrhagic events. Notably, nearly 60% of patients achieved a zero ABR under Emicizumab prophylaxis, yet 77.8% still reported pain within the last 12 months. This dissociation supports the hypothesis that, in the non‐factor era, haemophilia‐related pain may increasingly reflect structural joint sequelae and altered pain processing mechanisms rather than active haemorrhagic events. In the non‐factor era, pain may therefore increasingly reflect structural damage and complex central processing mechanisms rather than active haemorrhagic activity. This evolving landscape may represent a transitional phase in the epidemiology of haemophilia‐related pain, shifting from predominantly acute bleed‐associated pain to a chronic, multifactorial condition requiring different assessment and management paradigms. From a clinical perspective, these findings support the routine integration of structured pain assessment tools into haemophilia care, even in patients with well‐controlled bleeding. Systematic evaluation of pain interference and triggers may help to identify individuals requiring multidisciplinary interventions beyond haemostatic optimization.

However, several limitations of the study must be acknowledged. The small sample size and single‐centre design limit generalizability. The cross‐sectional design precludes causal inference or evaluation of temporal changes in pain following Emicizumab initiation. Pain data were self‐reported and may be influenced by recall bias. Although, standardized joint evaluations are routinely performed in our centre, arthropathy was analysed as a binary variable without severity stratification, which may have limited exploration of structure–symptom relationships. Finally, the absence of a comparator group treated with FVIII prophylaxis restricts direct comparisons between treatment modalities. Despite these limitations, this study provides real‐world evidence that pain persists as a multidimensional and clinically meaningful burden in adults with sHA receiving Emicizumab. By applying a structured, disease‐specific tool, we demonstrate that understanding contemporary haemophilia‐related pain requires moving beyond bleeding metrics and unidimensional intensity scales. Future longitudinal and multicentre studies are warranted to better delineate pain phenotypes, identify predictors of persistent pain, and develop tailored, patient‐centred interventions in the evolving therapeutic era of non‐factor prophylaxis.

## 5 Conclusions

In the contemporary non‐factor era, pain remains a prevalent and clinically relevant burden in adults with sHA despite effective prophylaxis with Emicizumab. Our findings suggest that haemophilia‐related pain is not exclusively bleeding‐driven but reflects a more complex and functionally impactful phenotype. These results underscore that optimal bleed control alone may not be sufficient to fully address pain‐related impairment, highlighting the importance of structured, multidimensional pain assessment and integrated management strategies in routine haemophilia care. Further longitudinal studies are warranted to better characterize persistent pain mechanisms and guide tailored interventions.

## Author Contributions

Conception and design of the study: Ilenia Lorenza Calcaterra and Matteo Di Minno. Acquisition of data: Sofia Donnarumma, Guido D'Errico, Ernesto Cimino and Paola Ciciola. Analysis and interpretation of data: Ilenia Lorenza Calcaterra and Carmine De Luca. Drafting the article: Ilenia Lorenza Calcaterra, Carmine De Luca and Chiara Caputo. Critical revision of the article for important intellectual content: Ilenia Lorenza Calcaterra and Matteo Di Minno. Final approval of the version to be submitted: Chiara Caputo, Carmine De Luca, Ernesto Cimino, Guido D'Errico, Ilenia Lorenza Calcaterra, Matteo Di Minno and Paola Ciciola.

## Declaration of AI‐Assisted Writing

During the preparation of this manuscript, the authors used *ChatGPT (OpenAI, GPT‐5.2, July 2025)* to assist in improving the language, structure, and clarity of the text. The AI was used exclusively for language polishing and editorial refinement. No content was generated without human supervision. All outputs were carefully reviewed, edited, and validated by the authors, who take full responsibility for the integrity and accuracy of the final manuscript.

## Funding

This study was conducted without direct external funding. Emicizumab treatment was part of routine clinical care and not sponsored by any pharmaceutical company. No commercial support influenced the design, analysis, or reporting of this study.

## Ethics Statement

This study was conducted in accordance with the Declaration of Helsinki and was approved by the local Ethics Committee of the University of Naples Federico II. All participants provided written informed consent prior to inclusion in the study.

## Conflicts of Interest

The authors declare no conflicts of interest related to this study. All authors have approved the final version.

## Data Availability

The data supporting the findings of this study are available from the corresponding author upon reasonable request. Due to privacy and ethical restrictions related to patient confidentiality, the data are not publicly available.
